# Assessment of Global and Local Alterations in Retinal Layer Thickness in Ins2 (Akita) Diabetic Mice by Spectral Domain Optical Coherence Tomography

**DOI:** 10.1155/2018/7253498

**Published:** 2018-02-20

**Authors:** Andrew W. Francis, Justin Wanek, Mahnaz Shahidi

**Affiliations:** ^1^Department of Ophthalmology, University of California San Francisco, San Francisco, CA, USA; ^2^Department of Ophthalmology and Visual Sciences, University of Illinois at Chicago, Chicago, IL, USA; ^3^Department of Ophthalmology, University of Southern California, Los Angeles, CA, USA

## Abstract

**Purpose/Aim:**

The Ins2 (Akita) mouse is a spontaneous diabetic mouse model with a heterozygous mutation in the insulin 2 gene that results in sustained hyperglycemia. The purpose of the study was to assess global and local retinal layer thickness alterations in Akita mice by analysis of spectral domain optical coherence tomography (SD-OCT) images.

**Materials and Methods:**

SD-OCT imaging was performed in Akita and wild-type mice at 12 and 24 weeks of age. Inner retinal thickness (IRT), outer retinal thickness (ORT), total retinal thickness (TRT), and photoreceptor outer segment length (OSL) were measured. Mean global thickness values were compared between Akita and wild-type mice. Local thickness variations in Akita mice were assessed based on normative values in wild-type mice.

**Results:**

Akita mice had higher blood glucose levels and lower body weights (*p* < 0.001). On average, IRT, ORT, and TRT were approximately 2% lower in Akita mice than in wild-type mice (*p* ≤ 0.02). In Akita mice, the percent difference between retinal areas with thickness below and above normative values for IRT, ORT, and TRT was 22%, 32%, and 38%, respectively.

**Conclusions:**

These findings support the use of the Akita mouse model to study the retinal neurodegenerative effects of hyperglycemia.

## 1. Introduction

Diabetic retinopathy (DR) is one of the most common causes of blindness worldwide [1–4]. The pathophysiology of this condition remains poorly understood, but its development and progression are associated with uncontrolled hyperglycemia, inflammation, neuronal dysfunction, and hypoxia. These various elements ultimately lead to the upregulation of growth factors, endothelial cell-specific mitogens, proteinases, and cytokines that cause pathologic alterations in the retinal architecture [5–9]. The application of experimental rodent models has improved our understanding of the disease process in DR, though there remains a need for rodent models that can replicate both the early and late clinical manifestations of DR [5–30]. Although transgenic, knockout, and toxin-induced rodent models have been studied, no rodent model to date has been able to accurately reproduce all vision-threatening features of DR including neovascularization, cystoid macular edema, and ischemia [25, 31–45].

Spectral domain optical coherence tomography (SD-OCT) imaging has become the standard of care in the diagnosis and management of DR [46–61]. Importantly, diabetic patients with minimal or no retinopathy demonstrate neurodegenerative alterations including retinal and choroidal thinning in SD-OCT images that often precede clinically identifiable DR [12, 13, 16, 17, 20, 21, 49, 50, 62–66]. It is of interest to investigate if retinal thickness alterations in patients with minimal to no DR can be reproduced in rodent models of diabetes.

The Ins2 (Akita) mouse is a spontaneous hyperglycemic mouse model of diabetes with an insulin 2 protein gene mutation that results in pancreatic *β*-islet cell death, hypoinsulinemia, and hyperglycemia starting at approximately four weeks of age [10, 36, 39, 42, 67]. Han et al. showed that the Akita mouse was similar in all aspects to the wild-type mouse before hyperglycemia occurred at approximately 4 weeks of life [36]. In their work, no differences between Akita and wild-type mice in vascular area, layer thickness on histology, or neurodegeneration were noted at 4 weeks. Significant increases in apoptotic retinal cells were observed in Akita mice as compared to wild-type mice at 6 and 9 months of age.

The majority of research studies investigating retinal alterations in the Akita mouse have employed ex vivo techniques such as histological sectioning, immunofluorescence, and trypsin digest models or in vivo methods including fluorescein angiography and confocal microscopy [10, 36, 37, 39, 42, 43, 68, 69]. There are a limited number of studies that have investigated retinal layer thickness alterations in the Akita mouse using SD-OCT imaging; however, the findings have been inconclusive. Specifically, retinal thickness was reported to be similar between Akita and wild-type mice by evaluating retinal thickness at localized regions using commercial software designed for human image analysis [37, 70]. Another study found inner and outer retinal layer thinning in Akita mice by using a manual image segmentation method [33]. In the present study, we assessed both global and local thickness changes due to sustained hyperglycemia in the Akita mouse model of diabetes by SD-OCT volume imaging and applying a semiautomated image segmentation method to map thickness of individual retinal cell layers.

## 2. Materials and Methods

### 2.1. Animals

Imaging was performed in aged-matched diabetic Akita (*N* = 22) and C57BL/6J wild-type (*N* = 22) mice at 12 weeks (Akita: *N* = 11, wild-type: *N* = 12) and 24 weeks (Akita: *N* = 11, wild-type: *N* = 10) of age. The mice were treated in compliance with the ARVO Statement for the Use of Animals in Ophthalmic and Vision Research. The mice were fed a Teklad diet (http://www.envigo.com/resources/data-sheets/7012-datasheet-0915.pdf), were exposed to 14 hr/10 hr of light/dark cycle, and housed in cages with a maximum number of 5 mice per cage. The mice were then anesthetized with intraperitoneal injections of ketamine (100 mg/kg) and xylazine (5 mg/kg). Additional injections were given to sustain anesthesia as needed. On the day of imaging, nonfasting blood glucose levels were measured from a drop of blood obtained by tail puncture with a commercially available blood glucometer (FreeStyle Lite, Abbott, Alameda, CA). Body weights of the mice were obtained. The femoral artery of each mouse was cannulated, and a catheter was attached. The mice were then placed in an animal holder, and their pupils were dilated with 2.5% phenylephrine and 1% tropicamide. Prior to SD-OCT imaging, 10% fluorescein sodium (5 mg/kg, AK-FLUOR®, Akorn, Decatur, IL) was administered through the femoral artery catheter for fluorescein angiography. SD-OCT image data was acquired in one eye in each mouse (left: *N* = 14; right: *N* = 30). During imaging, eyes were kept hydrated by frequent administration of eye drops. Image data collected from left eyes were transformed to orient all data to a right eye configuration.

### 2.2. Image Acquisition

A SD-OCT raster scan was obtained using a commercially available instrument (Spectralis; Heidelberg Engineering, Heidelberg, Germany). The raster scan consisted of 31 horizontal B-scans with a depth resolution of 3.9 *μ*m and 1536 A-scans per B-scan. The raster scan was acquired immediately nasal of the optic nerve head and covered a retinal area of approximately 30° × 25°. B-scans (16 in total) were averaged at each location using the instrument's eye tracker. Fluorescein angiography scanning laser ophthalmoscope (FA-SLO) images were acquired simultaneously during SD-OCT imaging to document the location of the SD-OCT raster scan on the fundus. Imaging was performed in both eyes, and images from one eye were selected for analysis based on image quality.

### 2.3. SD-OCT Segmentation

Our previously reported image segmentation software developed in MATLAB (Mathworks Inc., Natick, MA, USA) was used to identify four retinal cell layer interfaces in the SD-OCT B-scans [56, 61, 71]. Retinal cell layer interfaces were detected using graph theory and dynamic programming, based on a previous publication [72, 73]. In summary, a graph was generated for each SD-OCT B-scan with edge weights assigned based on the magnitude of the vertical gradients in the image, such that large gradients resulted in small weights. A horizontal path through the graph minimizing the total sum of the weights was obtained by Dijkstra's algorithm. Since retinal cell layer boundaries are typically characterized by large vertical intensity gradients, the path minimizing the weights of the graph defined a line separating two distinct retinal cell layers [72, 73].

**Figure 1:**
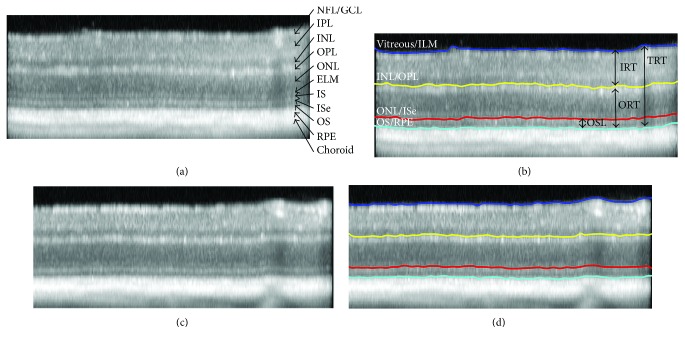
Examples of SD-OCT images acquired in wild-type (a and b) and Akita (c and d) mice. Individual retinal layers are labeled: nerve fiber layer (NFL), ganglion cell layer (GCL), inner plexiform layer (IPL), inner nuclear layer (INL), outer plexiform layer (OPL), outer nuclear layer (ONL), external limiting membrane (ELM), inner segments (IS), inner segment ellipsoid (ISe), outer segments (OS), retinal pigment epithelium (RPE), and choroid. Segmentation lines outlining 4 retinal cell layer interfaces are displayed, corresponding to the vitreous/internal limiting membrane (ILM); INL/OPL; ONL/ISe; and OS/RPE. Inner retinal thickness (IRT), outer retinal thickness (ORT), total retinal thickness (TRT), and photoreceptor outer segment length (OSL) are indicated by vertical arrows.

An example of a SD-OCT B-Scan image acquired in a wild-type mouse is shown in Figure 1(a). As labeled, the following individual retinal layers were visualized: combined nerve fiber layer (NFL) and ganglion cell layer (GCL), inner plexiform layer (IPL), inner nuclear layer (INL), outer plexiform layer (OPL), outer nuclear layer (ONL), external limiting membrane (ELM), photoreceptor inner segments (IS), photoreceptor inner segment ellipsoid (ISe), photoreceptor outer segments (OS), retinal pigment epithelium (RPE), and choroid. As shown in Figure 1(b), using a dedicated image segmentation method, four retinal layer interfaces were identified, namely, interfaces between (1) vitreous and internal limiting membrane (ILM), (2) INL and OPL, (3) ONL and ISe, and (4) OS and RPE.

Image segmentation was performed in a set order to find a unique path for these four retinal layer interfaces. First, the vitreous/ILM interface was identified since this was characterized by the largest positive vertical gradient (dark to bright transition) in the image and represented the lowest weighted path. Second, the OS/RPE boundary was found by restricting the graph search area to include only image regions below the vitreous/ILM path. Third, the INL/OPL cell interface was detected by limiting the graph to include only image regions between the vitreous/ILM and OS/RPE paths. Finally, the ONL/ISe interface was found by restricting the graph search area to include only image areas between the detected INL/OPL and OS/RPE interface.

If required, the operator was able to manually correct errors in the detected interfaces in the 31 SD-OCT B-scans. To correct segmentation errors, the operator would select a segmentation path that required modification and manually draw a revised line. The search area of the graph was then restricted to include only a small vertical image region around the manually drawn line, and a revised path for the cell layer interface was obtained by determining a new graph cut solution.

### 2.4. Thickness Mapping

Inner retinal thickness (IRT), outer retinal thickness (ORT), total retinal thickness (TRT), and photoreceptor outer segment length (OSL) were calculated from each SD-OCT B-scan, as indicated in Figure 1(b). IRT was defined as the depth separation between the vitreous/ILM and INL/OPL interfaces (IRT = NFL + GCL + IPL + INL). ORT was defined as the depth separation between the INL/OPL and OS/RPE interfaces (ORT = OPL + ONL + IS + ISe + OS). TRT was determined as the depth separation between the vitreous/ILM and OS/RPE interfaces (TRT = IRT + ORT). The OS/RPE interface was used as the retinal posterior boundary (instead of Bruch's membrane) due to the lack of a clear discrimination between the RPE and choroid in all mice. OSL was defined as the depth separation between the ONL/ISe and OS/RPE interfaces (OSL = ISe + OS). By compiling thickness data from all SD-OCT B-scans, IRT, ORT, TRT, and OSL maps were generated. In each thickness map, measurements that were in close vicinity to the optic disk (<1.5 disk diameter horizontally and vertically from optic disk center) were eliminated to avoid including alterations due to the normal optic disk depression.

### 2.5. Data Analysis

Global thickness measures (IRT, ORT, TRT, and OSL) were derived by averaging measurements within corresponding thickness maps. A two-way analysis of variance was used to evaluate the effects of condition (Akita and wild-type) and age (12 and 24 weeks) on mean thickness measures and body weight. In some diabetic mice, blood sugar measurements exceeded 600 mg/dL, the maximum level detectable by the glucometer. In these cases, a stand-in blood glucose value of 600 mg/dL was used and a nonparametric Mann-Whitney rank-sum test was performed to compare blood glucose between diabetic and wild-type mice. Statistical significance was accepted at *p* < 0.05.

Local thickness alterations in Akita mice were determined by assigning Z-scores to each pixel location in thickness maps based on the mean and standard deviation (SD) of thickness measurements calculated at the corresponding location from data in all wild-type mice. From each thickness map, the percentage of retinal area with Z-score values <−1, −1 to 1, or >1 was calculated. These percentages were averaged among all Akita mice, yielding a distribution that summarized the relative retinal area with thickness values below 1 SD (Z-score < −1), within 1 SD (−1 > Z-score < 1), and more than 1 SD (Z-score > 1) compared to normative values of wild-type mice. Using this method, we were able to determine the average percentage of retinal area in Akita mice that was thinner (Z-score < −1), thicker (Z-score > 1), or similar (−1 < Z-score < 1) to mean thickness in wild-type mice.

## 3. Results

Blood glucose measurements in Akita and wild-type mice are summarized in Table 1. In wild-type mice, blood glucose measurements were 151 ± 19 mg/dL (mean ± standard deviation, *N* = 12) at 12 weeks and 139 ± 19 mg/dL (*N* = 10) at 24 weeks of age. Blood glucose measurements in Akita mice were 269 mg/dL (*N* = 10) at 12 weeks and 380 mg/dL (*N* = 12) at 24 weeks of age. There was a significant difference in blood glucose levels between Akita and wild-type mice at both age groups (*p* < 0.001).

Mean body weight of wild-type mice was 28 ± 3 and 32 ± 3 grams at 12 and 24 weeks of age, respectively. Mean body weight of Akita mice was 24 ± 2 grams at both 12 weeks and 24 weeks of age. There was a significant age by condition interaction on body weight (*p* = 0.04). Wild-type mice had greater body weights at 24 weeks compared to 12 weeks of age (*p* = 0.008), while Akita mice had similar body weights at 12 and 24 weeks of age (*p* = 0.7). At both age groups, Akita mice had lower body weight than wild-type mice (*p* < 0.001).

The relative brightness of individual retinal layers in SD-OCT images in mice can be visualized in Figures 1(a) and 1(c), similar to normal reflectance properties in humans. In both Akita and wild-type mice, the NFL + GCL, IPL, OPL, ELM, ISe, and RPE were brighter than the INL, ONL, IS, and OS. Additionally, in the inner retina, both IPL and INL were distinctly visualized, while the NFL and GCL appeared as a single combined layer, in agreement with previous reports in mice [74, 75]. Similarly, interfaces between all outer retinal layers were clearly demarcated, except the interface between RPE and Bruch's membrane. As shown in Figures 1(b) and 1(d), the image segmentation method successfully detected retinal layer interfaces in both wild-type and Akita mice as outlined on SD-OCT images.

**Figure 2:**
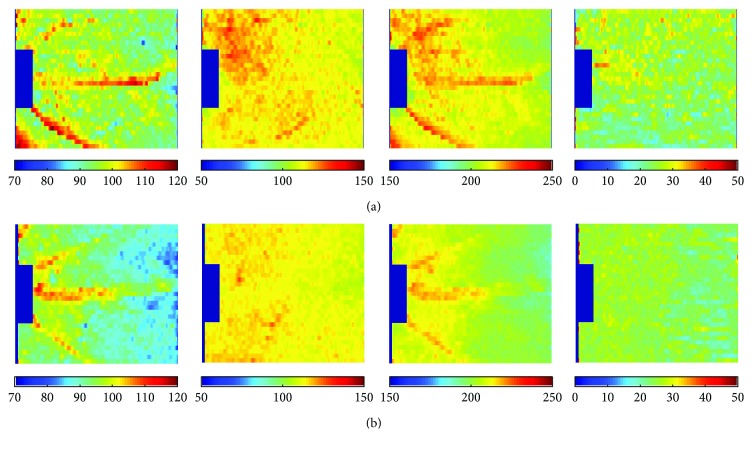
Thickness maps generated in (a) wild-type and (b) Akita mice. Left to right: inner retinal thickness (IRT), outer retinal thickness (ORT), total retinal thickness (TRT), and photoreceptor outer segment length (OSL). Color bars display thickness in microns.

Examples of IRT, ORT, TRT, and OSL thickness maps in one Akita and one wild-type mouse each are shown in Figure 2. These maps are visual color representations of variations in retinal thickness based on retinal geography for each separate layer. Areas with increased thickness are represented with warmer colors while areas with decreased thickness are represented with cooler colors. In both mice, major retinal vessels were visualized in the IRT and TRT maps. In addition, IRT, ORT, and TRT maps were higher near the optic disc in both wild-type and Akita mice, while the OSL maps were relatively uniform. Overall, decreased IRT, ORT, and TRT were present in maps of the Akita mouse compared to the wild-type mouse.

**Figure 3:**
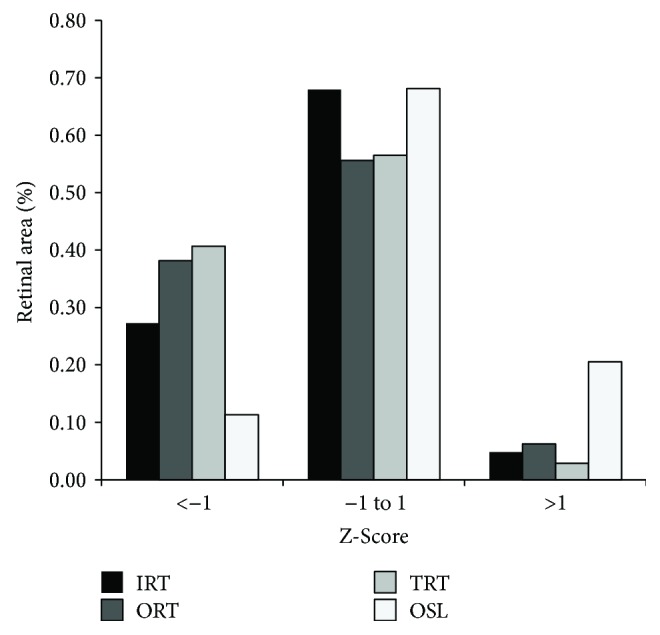
The percentage of retinal area plotted as a function of Z-score in Akita mice for inner retinal thickness (IRT), outer retinal thickness (ORT), total retinal thickness (TRT), and photoreceptor outer segment length (OSL).

The percentage of retinal areas plotted as a function of Z-score for IRT, ORT, TRT, and OSL is shown in Figure 3. The information represented in Figure 3 is a numerical interpretation of the information in Figure 2 with the Z-score as a calculated normative value of the mean. The percentage of retinal area with normal IRT, ORT, TRT, and OSL (−1 < Z-score < 1) was 68%, 56%, 57%, and 68%, respectively. The percentage of retinal area with reduced IRT, ORT, TRT, and OSL (Z-score < −1) was 27%, 38%, 41%, and 11%, respectively. The percentage of retinal area with increased IRT, ORT, TRT, and OSL (Z-score > 1) was 5%, 6%, 3%, and 21%, respectively. The difference between the percentage of retinal areas with reduced and increased IRT, ORT, and TRT was 22%, 32%, and 38%, respectively, indicating that in areas of abnormal retinal thickness, thinning occurred more frequently than thickening.

Compiled numerical IRT, ORT, TRT, and OSL data from all Akita and wild-type mice at 12 and 24 weeks of age are displayed in Table 2. In the wild-type mouse, the mean of IRT, ORT, and TRT was 94 ± 4 *μ*m, 114 ± 3 *μ*m, and 208 ± 6 *μ*m, respectively, at 12 weeks and 96 ± 6 *μ*m, 115 ± 2 *μ*m, and 211 ± 7 *μ*m, respectively, at 24 weeks. In the Akita mouse, the mean of IRT, ORT, and TRT was 92 ± 3 *μ*m, 112 ± 5 *μ*m, and 203 ± 8 *μ*m, respectively, at 12 weeks and 92 ± 4 *μ*m, 112 ± 2 *μ*m, and 204 ± 5 *μ*m, respectively, at 24 weeks. Mean OSL in both Akita and wild-type mice was 26 ± 2 *μ*m at 12 weeks, and 27 ± 2 and 26 ± 2, respectively, at 24 weeks. There was no significant effect of age or interaction between age and condition on IRT, ORT, TRT, or OSL (*p* ≥ 0.1). There was a significant effect of condition on IRT, ORT, and TRT. These thickness measures were significantly thinner in Akita mice compared to wild-type mice (*p* ≤ 0.02), while OSL was similar (*p* = 0.3).

## 4. Discussion

In this study, global and local alterations in retinal layer thickness were compared between Akita and wild-type mice by semiautomated segmentation of SD-OCT images obtained using a commercially available instrument. We report significant global thinning of the total retina due to a reduction in both inner and outer retinal layer thickness in Akita mice at 12 and 24 weeks of age and no significant alterations in photoreceptor outer segment length. Correspondingly, assessment of local thickness alterations showed thinning of inner and outer retinal layers in approximately one third of the evaluated retinal area. These findings indicate that significant neurodegeneration occurs in retinal cell layers of Akita mice as a result of sustained hyperglycemia. The Akita mouse holds promise as a suitable experimental surrogate to study the effects of uncontrolled hyperglycemia on retinal neurodegeneration that often precedes clinically identifiable DR.

In this study, retinal layer thickness was measured in Akita and wild-type mice by SD-OCT imaging, which has become an indispensable tool in the diagnosis and management of DR. Employing SD-OCT imaging allows longitudinal analysis of retinal thickness alterations, a clear advantage over histologic sectioning of retinal tissue. Additionally, in vivo evaluation of retinal thickness using SD-OCT imaging eliminates errors due to tissue shrinkage or damage that may occur during the processing of histologic sections [37]. Overall, SD-OCT imaging is a superior modality for in vivo retinal thickness assessment and is likely more sensitive and specific than ex vivo methods.

Measurements of retinal layer thickness in wild-type mice in the current study were consistent with previous reported values using SD-OCT. TRT, measured using the OS/RPE interface as the posterior retinal boundary, was similar to a previous study that used the same retinal interfaces [76] and in general agreement with previously reported TRT measurements that included RPE thickness [37, 74, 76–78]. Likewise, IRT and ORT in the current study were consistent with the previous studies [76, 79] and in general agreement with reported IRT measurements that included OPL thickness [74, 80]. Overall, the consistency between measurements in the current study and previously published values in wild-type mice serves as validation of retinal layer thickness obtained by our semiautomated image segmentation technique.

In the present study, TRT was reduced in Akita mice due to significant thinning of both inner and outer retinal cell layers. Previous studies have shown reductions in IRT and loss of ganglion cells in Akita mice using histopathology techniques [10, 42, 43, 45, 68]. Barber et al. [10] reported significant thinning of the IPL and INL as well as a reduction in the number of ganglion cell bodies in Akita mice compared to wild-type mice at 22 weeks of age. Smith et al. [81] reported that by 17 to 25 weeks of age, Akita mice demonstrated a 30% reduction in the IPL thickness, a 25% decrease in INL thickness, and a 30% reduction in ganglion cells. Rakoczy et al. [43] also reported significant thinning of the ganglion cell layer in Akita mice compared to wild-type mice. Our finding of reduced IRT and ORT by in vivo imaging of Akita mice is in agreement with a previous study that used a similar imaging modality [33].

Other studies utilizing SD-OCT imaging have reported no differences in TRT between Akita and wild-type mice [37, 70]. The discrepancy between these findings may be attributed to variations in sample size, imaging region, and image segmentation method. Since identification of Bruch's membrane is more challenging in mice than in humans, the detection of this interface with the use of commercially available automated segmentation software may be less accurate and more variable compared to the current technique which used the OS/RPE boundary for calculation of TRT.

Clinical studies in human type 1 diabetics with minimal or no retinopathy report retinal thinning, similar to the findings in the Akita mouse, suggesting this model may be a suitable surrogate for the study of neurodegenerative alterations in the retina that precede clinically identifiable DR [12, 13, 17, 63, 82–84]. Biallosterski et al. [49] compared retinal thickness measurements of patients with and without type 1 diabetes mellitus and found that the mean retinal thickness in the pericentral area was thinner in diabetic patients. Asefzadeh et al. [48] reported that the degree of macular and foveal thinning correlated with duration of disease in diabetic subjects with minimal or no DR. Additionally, van Dijk et al. [12, 64] found significant thinning of the inner retinal layers, specifically the GCL, IPL, and INL, in type 1 diabetic patients with minimal retinopathy as compared to healthy controls. Taken collectively, these findings indicate that neurodegenerative alterations may cause thinning of the inner retinal layers, a potential hallmark of subclinical DR. If true, the ability of the Akita mouse to reproduce reduced retinal thickness validates its use as an appropriate model to study early neurodegenerative changes in the human retina.

The etiology of retinal thickness changes reported in this study is likely due to vascular, inflammatory, degenerative, and neural abnormalities that occur as a result of sustained hyperglycemia. Vascular incompetency demonstrated by acellular capillaries, decreased choroidal blood flow, imbalanced pro- and antiangiogenic factors including vascular endothelial growth factor, and leakage has been reported in this model [31, 36, 41, 42]. Inflammatory changes, including the presence of reactive microglial cells, were shown to be present as early as eight weeks of age [10]. Degenerative changes including pericyte ghosts, basement membrane thickening, decreased a- and b-wave amplitudes, dendrite remodeling, and increased apoptosis of retinal ganglion cells have been previously reported [10, 17, 32, 36, 69]. In addition, neural abnormalities such as morphological alterations in astrocytes, decreased cholinergic and dopaminergic amacrine cells, and increased inflammatory leukocytes have also been observed in this model [10, 17, 68, 69]. Further in vivo studies utilizing high-resolution imaging systems are needed to determine the interplay of these factors that contribute to decreased retinal thickness in this experimental model.

## 5. Conclusion

In summary, significant thinning of inner, outer, and total retinal thickness was reported in the Ins2 (Akita) diabetic mouse using SD-OCT imaging. These findings support the use of the Akita mouse as an experimental model to study the effects of hyperglycemia on retinal thickness.

## Figures and Tables

**Table 1:** Blood glucose measurements of Akita and wild-type mice at 12 and 24 weeks of age.

Variable	Age (weeks)	Akita	Wild-type	*p* value
Blood glucose (mg/dL)	12	≥269	151 ± 19	<0.001^∗^
	24	≥380	139 ± 19	<0.001^∗^

^∗^Mann-Whitney rank-sum test.

**Table 2:** Inner retinal thickness (IRT), outer retinal thickness (ORT), total retinal thickness (TRT), and photoreceptor outer segment length (OSL) in Akita and wild-type mice at 12 and 24 weeks of age.

Variable (microns)	Age (weeks)	Akita	Wild-type	% difference	*p* value		
					Condition	Age	Condition ∗ age
IRT	12	92 ± 3	94 ± 4	−2.13	0.02	0.4	0.8
	24	92 ± 4	96 ± 6	−4.17			

ORT	12	112 ± 5	114 ± 3	−1.75	0.008	0.3	0.4
	24	112 ± 2	115 ± 2	−2.61			

TRT	12	203 ± 8	208 ± 6	−2.40	0.004	0.3	0.6
	24	204 ± 5	211 ± 7	−3.32			

OSL	12	26 ± 2	26 ± 2	0.00	0.3	0.1	1.0
	24	27 ± 2	26 ± 2	+3.85			
